# The Role of Semaphorin 4D in Bone Remodeling and Cancer Metastasis

**DOI:** 10.3389/fendo.2018.00322

**Published:** 2018-06-19

**Authors:** Konstantinos Lontos, Juraj Adamik, Anastasia Tsagianni, Deborah L. Galson, John M. Chirgwin, Attaya Suvannasankha

**Affiliations:** ^1^Hematology-Oncology Division, Department of Medicine, UPMC Hillman Cancer Center, McGowan Institute for Regenerative Medicine, University of Pittsburgh, Pittsburgh, PA, United States; ^2^Department of Medicine, University of Pittsburgh Medical Center, Pittsburgh, PA, United States; ^3^Hematology and Oncology Division, Department of Medicine, Indiana University School of Medicine, Richard L. Roudebush VA Medical Center, Indianapolis, IN, United States

**Keywords:** semaphorin 4D, Sema4D, Plexin-B1, plexin, osteoclasts, osteoblasts, cancer

## Abstract

Semaphorin 4D (Sema4D; CD100) is a transmembrane homodimer 150-kDa glycoprotein member of the Semaphorin family. Semaphorins were first identified as chemorepellants that guide neural axon growth. Sema4D also possesses immune regulatory activity. Recent data suggest other Sema4D functions: inactivation of platelets, stimulation of angiogenesis, and regulation of bone formation. Sema4D is a coupling factor expressed on osteoclasts that inhibits osteoblast differentiation. Blocking Sema4D may, therefore, be anabolic for bone. Sema4D and its receptor Plexin-B1 are commonly dysregulated in cancers, suggesting roles in cancer progression, invasion, tumor angiogenesis, and skeletal metastasis. This review focuses on Sema4D in bone and cancer biology and the molecular pathways involved, particularly Sema4D–Plexin-B1 signaling crosstalk between cancer cells and the bone marrow microenvironment—pertinent areas since a humanized Sema4D-neutralizing antibody is now in early phase clinical trials in cancers and neurological disorders.

## Introduction

Semaphorin 4D (Sema4D; also known as CD100), is a member of class IV of the Semaphorin protein family, with established functions as an immune regulator. This review focuses on additional, emerging roles of Sema4D in bone biology and cancer bone metastases. Recent pivotal findings support the pertinence of Sema4D in bone and cancers: (1) Negishi-Koga et al. (1) identified Sema4D as a major coupling factor expressed on osteoclasts that inhibits osteoblast differentiation. They found that mice with a global knockout of *Sema4d* had increased bone volume. (2) Terpos et al. (2) reported increased soluble Sema4D in serum and bone marrow plasma of patients with myeloma, a bone marrow cancer with uncoupled osteoclast activation and osteoblast suppression, compared to controls. (3) Sema4D and its primary receptor Plexin-B1 are commonly overexpressed in cancers. (4) Yang et al. (3) found that shRNA knockdown of Sema4D in MDA-MB-231 breast cancer cells decreased bone metastases in a standard xenograft model. (5) A humanized antibody that neutralizes Sema4D has shown antitumor activity in animal models and is under clinical testing in early phase clinical trials (4).

## SEMA4D Structure and Role in Human Physiology

Semaphorins form a highly conserved family of proteins that contain a signature amino-terminal sema domain. The semaphorin family contains more than 20 genes divided into seven classes, of which classes III–VII are expressed in vertebrates. They have diverse roles in human biology including regulation of tumor growth and metastasis, angiogenesis, axonal guidance, bone formation, tissue regeneration, and autoimmunity (5).

Sema4D belongs to class IV of the Semaphorin family. In addition to the signature sema domain, the C-terminal region of Sema4D includes an IgG-like domain, a transmembrane domain, and a short cytoplasmic tail that contains one tyrosine phosphorylation site and multiple sites for serine–threonine phosphorylation. Membrane-bound Sema4D forms a stable homodimer *via* a disulfide bond between cysteines 679 within the sema domain. Proteolytic shedding of Sema4D by membrane-type 1-matrix metalloproteinase (MT1-MMP/MMP14) gives rise to soluble, dimeric Sema4D (sSema4D) (6). Both membrane-bound and soluble Sema4D can activate Plexin-B1 signaling.

Sema4D is expressed by many tissues including brain, kidney, and heart. However, Sema4D knockout mice have immune defects without other obvious organ dysfunctions, suggesting that its major role is in immune regulation. Sema4D is expressed strongly by resting T cells and weakly on B and antigen-presenting cells. Expression is increased upon cellular activation (7). Engagement of Sema4D enhances its association with the membrane protein tyrosine phosphatase CD45, which is expressed broadly in hematopoietic cells (8). The complex becomes active and recruits further proteins to sustain B and T cell activation and aggregation.

Most work on Sema4D function has focused on its role as a ligand in soluble form after proteolytic shedding. Several receptors for Sema4D have been identified, including C-type lectin protein CD72, and three members of the plexin family; Plexin-B1, Plexin-B2, and Plexin-C1 (9–12). Target cells express different receptors, leading to a broad variety of potential responses to Sema4D in different tissues. CD72 is the main Sema4D receptor on immune cells, although monocytes and immature dendritic cells require Plexin-C1 and Plexin-B1, respectively. Plexins-B1 and -B2 mediate the Sema4D responses on non-immune cells. CD72 (also known as Lyb-2) is a 45-kDa type II transmembrane protein of the C-type lectin family (13) which is expressed throughout B-cell differentiation (14). The CD72 cytoplasmic domain contains two immune-receptor tyrosine-based inhibition motifs that recruit the tyrosine phosphatase SHP-1, resulting in inhibition of src family kinases and JNK and B cell inhibition (15). Sema4D engagement of CD72 triggers tyrosine dephosphorylation of CD72, leading to SHP-1 dissociation (10), thereby relieving CD72-mediated B cell inhibition. Since Sema4D and CD72 are expressed preferentially on T and B cell, respectively, their interaction couples T and B cells to dial the immune reaction up or down. Dendritic cells, macrophages, and some subpopulations of T cells express CD72 (16). Sema4D may, therefore, play an additional role in T cell communication *via* these other immune cells.

Plexins are transmembrane proteins with a sema ligand-binding domain in their extracellular domain. Upon ligand binding, Plexin-B1 and Plexin-B2 extracellular domains undergo proteolysis by subtilisin-like proprotein convertases to further increase their affinity for Sema4D (17). The highly conserved cytoplasmic region of plexins is devoid of enzymatic activity, but it can interact, directly or indirectly, with small G proteins for various functions (18). Transduction cascades downstream of the Sema4D/Plexin-B1 complex vary, dependent on the different membrane proteins and G proteins recruited to the complex.

**Figure 1:**
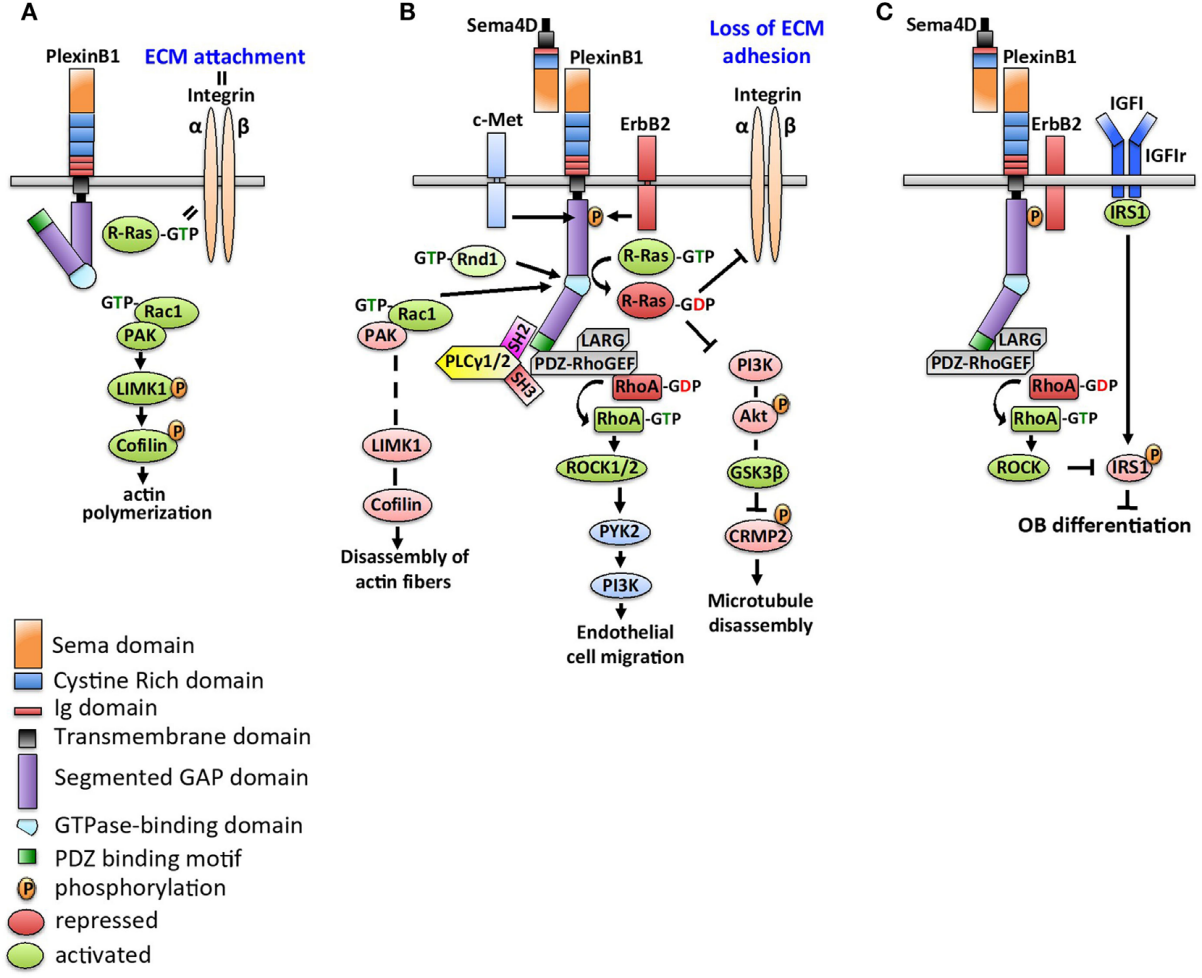
Schematics of Sema4D/Plexin-B1 signaling. **(A)** In the absence of Sema4D, the cytoplasmic tail of Plexin-B1 is in an inactive conformation. R-Ras is in a GTP-bound state to collaborate with integrin and control cellular adhesion to the extracellular matrix (ECM). With Plexin-B1 in an inactive state, Rac activates p21-activated kinase (PAK) to trigger LIMK1 signaling and downstream phosphorylation of cofilin, which results in increased actin polymerization and microtubule assembly. **(B)** Sema4D binding to Plexin-B1 alters the Plexin-B1 conformation, recruits Rac1 to the complex, and inhibits activation of the PAK–LIMK1–cofilin signaling cascade. In addition, R-Ras recruitment to the Sema4D/Plexin-B1 complex inhibits its ability to regulate integrin-mediated activation, resulting in increased cell motility. Deactivation of R-Ras further decreases PI3K-Akt GSK3β pathway, which leads to deactivation of CRMP-2 and subsequent microtubule disassembly. In an ErbB2-dependent signaling step, PLCγ1/2 is recruited to Plexin-B1 *via* its SH2 domain. Structural interaction of the PLCγ1/2 SH3 domain triggers PDZ-RhoGEF/LARG complex, resulting in RhoA activation. Active GTP-RhoA acts on Raf and Rho-associated kinases (ROCK1/2), which in turn stimulate PYK2 to induce cell invasiveness and migration. **(C)** Sema4D-Plexin-B1 signaling inhibits osteoblast differentiation. Plexin-B1/ErbB2-dependent RhoA activation stimulates activation of downstream kinase ROCK, which phosphorylates the insulin receptor substrate 1 (IRS1). This causes suppression of insulin like growth factor (IGF1)-dependent signaling and blocks osteoblast differentiation.

Figure 1 details downstream signaling of Sema4D/Plexin-B1. Without engagement with Sema4D, the cytoplasmic tail of Plexin-B1 is in an inactive conformation. R-Ras is in a GTP-bound state and activates membrane integrin to control cellular adhesion to the extracellular matrix. Rac is not bound to Plexin-B1 and promotes activation of p21-activated kinase (PAK) to activate LIMK1 and cofilin to increase actin polymerization and microtubule assembly (19). Binding of Sema4D to Plexin-B1 alters Plexin-B1 conformation allowing recruitment of Rac1, which sequesters it from the PAK-LIMK1-cofilin signaling cascade, causing disassembly of actin fibers (20). Initiation of PlexinB1-GAP activity inhibits R-Ras-mediated integrin activation, which blocks cell adhesion and increase cell motility and invasion (21). Deactivation of R-Ras further decreases the PI3K–Akt–GSK3β pathway, which leads to deactivation of CRMP-2 and subsequent microtubule disassembly (22).

Whether Sema4D/Plexin-B1 complexes activate or inhibit downstream GTPases also depends on their interactions with specific receptor tyrosine kinases: ErbB-2 or c-Met (23). In ErbB2 expressing cells, binding of Sema4D to Plexin-B1 activates the intrinsic tyrosine kinase activity of ErbB-2, resulting in the phosphorylation of both Plexin-B1 and ErbB-2 (24), generating docking sites on Plexin-B1 for the SH2 domains of 1-phosphatidylinositol-4,5-bisphosphate phosphodiesterase gamma-1/2 (PLCγ1/2) (25). The SH3 domain of the recruited PLCγ1/2 activates the guanine nucleotide-exchange factors (GEFs) LARG (leukemia-associated RhoGEF) and PRG (PDZ-RhoGEF) that constitutively bind to the PDZ binding site of the carboxyl-terminal sequence of Plexin-B1 (26). Activated RhoGEFs LARG and PRG mediate activation of the small GTPase RhoA, which cooperates with Ras to activate the serine/threonine kinases Raf and Rho-associated kinases (e.g., ROCK1/2) to stimulate various pathways, including mitogen-activated phosphokinases (MAPKs) ERK1/2 and p38, protein kinases (PI3/Akt—phosphatidylinositol 3′-kinase) (27). Downstream activation of MAPKs and PI3K control dendritic and axonal morphogenesis by differentially regulating branching and extension (27). On the other hand, Sema4D/Plexin-B1 can interact with Rnd1 to downregulate the GTPase activity of R-Ras, inducing growth cone collapse in hippocampal neurons (28). Plexin-B1 also binds and competes for activated Rho GTPase Rac, thus preventing Rac from activating its downstream effector PAK to initiate actin polymerization. In endothelial cells, Sema4D/Plexin-B1 signals stimulate PI3K-Akt to activate PYK2 and Src to control endothelial cell migration (29). c-Met (HGF receptor tyrosine kinase) and macrophage stimulating protein (MSP, encoded by the Ron gene) are members of the Scatter Factor Receptors family. They share structural homology with Plexin-B1 (30, 31). In cells with c-Met expression but absent ErbB2, Sema4D/Plexin-B1 recruits and activates c-Met or Ron kinases to regulate cell motility and invasiveness. In normal fibroblasts, phosphorylation of c-Met creates a docking site for growth factor receptor bound-2 (Grb2), which then interacts with p190 RhoGAP and inactivates RhoA, causing inhibition of fibroblast motility (32).

## SEMA4D in Bone Physiology

Successful crystallization of Sema4D and solution of its structure led to a recognition of the homology between the Sema4D homodimer and the αVβ3 integrin heterodimer, the first clue to a role for Sema4D in bone biology (33). αVβ3 integrin is an osteoclast regulator, and β3 integrin knockout mice become progressively osteosclerotic with age due to dysfunctional osteoclasts that fail to polarize correctly and display abnormal ruffled membranes (34).

Two groups reported an increased bone mass in Sema4D knockout animals and the expression of Sema4D in osteoclasts but not osteoblasts (1, 35). Negishi-Koga and coworkers comprehensively surveyed osteoclasts and osteoblasts for expression of candidate molecules to couple bone formation to resorption, including the members of the ephrin, netrin, semaphorin, and slit gene families. They found that only Sema4D was highly expressed in RANKL-activated osteoclasts. Utilizing a soluble Fc receptor-Sema4D fusion protein, they evaluated directly the effects of the Sema4D extracellular domain on osteoblasts. Fc-Sema4D inhibited the osteoblast differentiation markers alkaline phosphatase and osteocalcin, as well as the formation of mineralized nodules in culture, without a change in osteoblast proliferation. Co-immunoprecipitation confirmed a complex of Fc-Sema4D with Plexin-B1, which is highly expressed in osteoblasts. This complex leads to ErbB2 phosphorylation and downstream activation of RhoA. *Plexin*^−/−^ animals, as well as mice expressing an osteoblast-targeted dominant negative RhoA, had a high bone mass due to enhanced osteoblastic bone formation, recapitulating the bone phenotype of the *Sema4d*^−/−^ mice. A recent study used optoPlexin (optogenetic activation of Plexin-B1) in osteoblasts to show that Plexin-B1 activation results in retraction of the leading edge and induces distal membrane protrusions, causing the osteoblasts to migrate. Thus, osteoclast-produced Sema4D may cause repulsion of osteoblasts *via* activation of both the RhoA and rasGTP pathways (36). Overall, these findings support the function of the Sema4D/Plexin-B1/RhoA axis in osteoblast inhibition by osteoclasts (1). However, Sema4D’s regulation of bone mass may be more complicated. Dacquin et al (35) noted that the increased bone mass phenotype in *Sema4d*^−/−^ mice primarily occurred in mature female mice. The increased bone mass phenotype in *Sema4d*^−/−^ mice was not reversed after a transplantation of bone marrow cells from wild-type mice, as a source of new osteoclasts. *Sema4d*^−/−^ mice have impaired ovarian function, small litter size, and decreased hypothalamic gonadotropin-hormone releasing hormone. The authors propose an indirect mechanism by which Sema4D regulates bone mass *via* the hypothalamic–pituitary–ovarian axis (35).

Serum Sema4D has been explored as a biomarker in osteoporosis, with conflicting results to date, likely reflecting different patient populations, the non-randomized nature of the trials and different time points for Sema4D analysis. In postmenopausal women, serum Sema4D was higher among those with osteoporosis (37). However, in an open label study of osteoporotic women receiving the osteoclast-targeting agents zoledronic acid or RANKL monoclonal antibody denosumab or the bone-anabolic agent teriparatide (PTH1-34), serum Sema4D decreased after 3 months of teriparatide treatment, while it increased with denosumab and zoledronic acid (38). There was no attempt to correlate the Sema4D levels with degree of osteoporosis, and serum Sema4D was assayed at only one time point, not allowing a determination of kinetics, which may differ among treatment groups.

Congenital defects due to mutation or loss of Sema4D have been little studied in humans. Segmental copy number loss in the region of the Sema4D gene was seen in one third of patients with acetabular dysplasia, which increases risk of osteoarthritis, but a causal relationship was not explored (39).

Sema4D has been implicated in bone and joint inflammation. In rheumatoid arthritis (RA), Sema4D was elevated in both serum and synovial fluid from RA patients, and disease activity markers were correlated with serum Sema4D levels. Sema4D-expressing cells also accumulated in RA synovium, and sSema4D-induced tumor necrosis factor α (TNFα) and interleukin-6 (IL-6) production from CD14+ monocytes (40). Movila and group explored treatment-related osteonecrosis of the jaw and showed increased Sema4D-expressing γδ-T cells in bone lesions, which were decreased by anti-Sema4D antibody (41). In osteoarthritis, bone tissue had low Plexin-B1 expression compared to age- and gender-matched cadaveric control bones, indicating that the loss of Sema4D/Plexin-B1 inhibition of osteoblast activity may lead to an increased bone volume fraction and decreased bone matrix mineralization, contributing to osteoarthritis (42).

Elevated Sema4D is observed in diseases unrelated to the musculoskeletal systems, including hemorrhagic fever with renal syndrome (43) and autoimmune diseases (44). Inflammation and increased shedding from peripheral blood mononuclear cells are possible sources. Serum Sema4D is also increased in non-inflammatory condition such as atrial fibrillation (45). The value of sSema4D as a biomarker in some of these diseases may warrant further investigation.

## SEMA4D in Cancer

While other semaphorins such as Sema3B and 3F are tumor suppressors, Sema4D promotes tumor growth (46, 47). Sema4D and Plexin-B1 are abnormally expressed in various cancers and have been associated with invasive phenotypes and poor prognosis. The mechanisms by which Sema4D confers these features are complex and involve both tumor cells and their microenvironment. While not directly linked to malignant transformation, Sema4D/Plexin-B1 signaling contributes to many critical aspects of cancer progression, including proliferation, invasion, angiogenesis, immune escape, and metastasis. We summarize the role of Sema4D/Plexin-B1 in cancers based on overexpression of Sema4D or Plexin-B1 by the cancer cells or by other cells in the tumor microenvironment including immune cells, and the role of Sema4D/Plexin-B1 in distant metastasis.

Because of its known role in lymphoid cells, Sema4D expression in cancer was first evaluated in lymphoma and lymphoblastic leukemia. T cell lymphomas universally express Sema4D, while only a minority of B cell lymphomas are Sema4D positive (48), paralleling the expression of Sema4D by normal T and B cells. In chronic lymphocytic leukemia (CLL), CD38 is a known negative predictor of survival. CD38 binds to (49) stromal cells expressing CD31, leading to relocalization of Sema4D, facilitating its binding to Plexin-B1 on bone marrow cells. The increased expression of Plexin-B1 in stromal cells, follicular dendritic cells, and activated T-cells, enhances the complex interplay of CD38/CD31 and Sema4D/Plexin-B1 to sustain CLL growth (50, 51).

Sema4D is highly expressed by many solid tumors including prostate, glioma, lung, ovarian, sarcoma, and cutaneous squamous cell carcinoma. Expression is correlated with tumor aggressiveness and poor prognosis, but controversial data exist for some tumors. In an array of 888 genes, Sema4D mRNA was highest in early stage breast cancer compared to normal tissue and downregulated in advanced disease (52). Jiang and coworkers confirmed high Sema4D protein in breast cancer cell lines compared to normal breast epithelial cells. In addition, knockdown of Sema4D by shRNA inhibit breast cancer proliferation and tumor growth in xenografts (49). Malik et al., however, showed an opposite result of decreased Sema4D, Plexin-B1 and -B2 protein in primary breast tumors of patients who subsequently developed local recurrence, compared to the patients who remained disease-free (53). These studies used whole tumor tissues and may be confounded by different cell types within the tissue, since other cells in the microenvironment, including endothelial cells and macrophages, also express Sema4D. In whole-blood RNA, Sema4D was identified as one of six RNAs strongly predicting shorter survival among patients with castrate-resistant prostate cancer (54). Most data on Sema4D and clinical outcomes are based on small sample sizes with subjects receiving different treatments and not controlled for other prognostic markers. Prognostic value of Sema4D requires further validation in larger cohorts of patients, controlled for treatment types and other variables.

Overexpression of Plexin-B1 has been reported in skin, prostate and pancreatic cancers, and sarcoma (55–57). It correlates with lymph node metastasis, distant metastasis, and poor prognosis in patients with pancreas cancer (56). Plexin-B1 activation increases phosphorylation and translocation into the nucleus of the androgen receptor (AR), leading to activation of AR-regulated genes, which could play a role in castration resistance in prostate cancer (58). However, Rody et al. showed a rather opposite finding in breast cancer, in which loss of Plexin-B1 identifies a subgroup of estrogen receptor expressing breast cancer with high proliferative rate and hormone resistance (59). Cancer cell lines that express higher levels of Plexin-B1 exhibit increased perineural invasion. The mechanism is proposed to be the attraction of the cells due to Sema4D production from the nerves. Interestingly, higher nerve density in tumors expressing Sema4D (60) suggests that these tumors may use nerve-secreted factors for growth, pointing to a possible role of Sema4D in cancer pain.

While no Sema4D mutation has been reported in human cancers, Plexin-B1 mutations and copy number changes are noted commonly in various cancers, including melanomas, pancreas, breast, and prostate cancers (58, 61, 62). Thirteen somatic missense mutations in the cytoplasmic domain of Plexin-B1 were found in 46% of prostate cancers. Mutational hotspots mapped to the Rho GTPase binding domain in the cytoplasmic region of the receptor, causing loss of Rac and R-Ras binding and R-RasGAP activity. This resulted in an increase in cell motility, invasion, and adhesion, and could explain the metastatic phenotype (61). In some cancers, however, Plexin-B1 acts as a tumor suppressor. For example, Plexin-B1 is lost in deep and metastatic melanomas. Introducing Plexin-B1 into melanoma cells suppresses c-Met and hence proliferative responses to HGF. Increased Plexin-B1 confers resistance to cisplatin (63). A similar finding was noted in clear cell carcinoma (64). Sema4D/Plexin-B1 responses may vary among different cell lines of the same tumor type. Sema4D/Plexin-B1 increased the proliferative and invasive potential of LNCaP prostate cancer cells through the activation of ErbB2 and Akt, but decreased the motility and proliferation of PC3 prostate cells (65).

Different phenotypes of Sema4D/Plexin-B1-expressing tumors may depend on the expression of its partner proteins ErbB2 and c-Met. Since c-Met is one of the most commonly deregulated oncogene in cancers, its collaboration with Sema4D/Plexin-B1 is an alternative pathway to promote tumor invasion (66). Constitutive activation of Met in tumor cells with high Plexin-B1 can occur in the absence of Sema4D (66). T lymphoma invasion and metastasis 1 (Tiam1) is another Rac-specific guanine nucleotide-exchange factor that is activated by Sema4D/Plexin-B1 to stimulate Rac and promote proliferation, invasion, and metastasis in oral squamous cell carcinoma (67). In cancer cells that express both Sema4D and Plexin-B1, the pair could function in an autocrine/paracrine manner, although this requires future study.

In addition to direct proliferative actions in cancer cells, Sema4D/Plexin-B1 abnormalities within the tumor niche support cancer progression by promoting angiogenesis. Sema4D/Plexin-B1 function in angiogenesis was first described by Basile and coworkers (68), who showed that recombinant Sema4D-induced chemotaxis of endothelial cells, *in vitro* tubulogenesis and enhanced blood vessel formation in an *in vivo* mouse model. Sema4D/Plexin-B1 phosphorylation of c-Met promotes angiogenesis in a non-redundant manner from HGF (69). Sema4D can also stimulate angiogenesis *via* a c-Met-independent pathway through recruitment of PDZ-RhoGEF and LARG to the Sema4D/Plexin-B1 complex leading to Rho pathway activation, followed by downstream AKT and NF-κB activation and increased expression of proangiogenic IL-8 (70). Hypoxia induces Sema4D in a HIF-1-dependent manner. Sema4D then cooperates with VEGF to promote tumor growth and vascularity (71, 72). In addition, tumor-secreted Sema4D increases endothelial expression of platelet-derived growth factor-B and angiopoietin-like protein 4, which promote endothelial proliferation and vascular permeability (73). Lentiviral overexpression of Sema4D in colorectal cancer cell lines caused a proangiogenic response regardless of VEGF status (74). Sema4D may also be a biomarker for tumor angiogenesis, since its expression in ovarian cancer correlates with HIF-1, VEGF, and poor prognosis. Sema4D inhibition causes dissociation of endothelial cells from pericytes, a crucial step for successful antiangiogenic therapy (75). Both VEGF and Sema4D may cooperate in a multi-step process to reorganize the vasculature within the malignant niche. Tumor cells increase their Sema4D expression as an escape mechanism from anti-VEGF treatment (75). Concurrent targeting of VEGF and Sema4D may have additive or synergistic antiangiogenic effects. Sema4D may also support lymphangiogenesis, since Sema4D targeting with neutralizing antibody or shRNA suppressed VEGF-C and VEGF-D, key factors for lymphangiogenesis (76).

The role of Sema4D in immune regulation supports the importance of dysregulated Sema4D in immune escape of cancer cells. In a head and neck cancer model, tumor-secreted Sema4D promoted the expansion of myeloid-derived suppressor cells, which inhibit T-cell functions (77). Sema4D affects both the activities of immune cells and their recruitment to the tumor microenvironment. Delaire and coworkers noted that Sema4D inhibited both spontaneous and chemokine-induced migration of human monocytes (78). Strong expression of Sema4D at the invasive margins of actively growing tumors changed the infiltration and distribution of leukocytes within the tumor microenvironment. Neutralization of Sema4D by blocking antibodies disrupted this gradient of expression and enhanced recruitment of activated monocytes and lymphocytes into the tumor. This shifted the balance of cells and cytokines in a pro-inflammatory and antitumorigenic direction and was associated with durable tumor rejection in murine Colon26 and ERBB2^+^ mammary carcinoma models (4). These functions are at odds with the known roles of Sema4D in T and B cell activation. The dual nature of Sema4D between pro and antitumor action may depend on subsets of immune cells in the tumor niche, which in turn may depend on the plasticity of macrophages and T cells within the tumor. Classical activation of macrophages with interferon γ (M1) promotes the differentiation of cytotoxic T cells, which can improve antigen phagocytosis. However, alternative pathway of macrophage activation by IL-4, IL-14, or LPS gives rise to M2 macrophages. Tumor-associated monocytes are M2 and communicate effectively with regulatory T cells (Treg) to suppress antigen recognition and promote an inflammatory tumor microenvironment, angiogenesis, and tumor progression (79). In tumors with high M2 macrophages and Tregs, Sema4D may contribute to immune suppression, even though in normal physiology, Sema4D is required for T and B cell function.

Sema4D may promote metastasis at distant sites. Many cancer cells, including head and neck squamous cell carcinoma lines, express the membrane-tethered collagenase, MT1-MMP which cleaves Sema4D. sSema4D could thus promote angiogenesis and cell migration both locally and at distant sites, thereby promoting metastasis (80).

## SEMA4D in the Bone Marrow Metastatic Niche

The involvement of Sema4D in bone biology and cancer progression suggests a role in bone metastasis. Bone is the most common site of distant metastases for prostate and breast cancers. Bone metastases are driven by complex interactions between cancer cells, bone marrow cells, and bone cells, often leading to increased osteoclast and suppressed osteoblast activities. Even in osteosclerotic metastasis, occurring in prostate and some breast cancers, the newly formed bone is poorly organized, and osteoclasts remain activated, leading to loss of bone quality. Osteolysis with osteoblast suppression is a hallmark of multiple myeloma, an incurable blood cancer that originates within bone (81, 82). Current treatments are limited to osteoclast-targeting agents, which provide palliative benefit with marginal effect on tumor control. Zoledronic acid modestly prevents bone metastases in breast and prostate cancer xenograft models, but its benefit as a bone metastasis prevention is limited to postmenopausal women (83). No survival advantage was seen in premenopausal women or men with prostate cancers. This evidence suggests that osteoclast targeting alone is inadequate for tumor control.

Sema4D is a coupling molecule of osteoclasts and osteoblasts (1). In the tumor niche, Sema4D from the tumor cells and activated osteoclasts inhibits osteoblast differentiation, while inducing IL-8 secretion to further promote osteoclast proliferation and activity (70). Because soluble Sema4D can reach distant bone sites, it could hypothetically prime the bone niche to support future metastasis. Since tumor cells with high Sema4D also have high motility and invasiveness and Sema4D promotes angiogenesis, Sema4D could contribute to metastasis at both at the primary tumor site and distant sites. This notion is supported by a decrease in skeletal metastasis when Sema4D was knocked down using shRNA in the MDA-MB-231 breast cancer model (3).

Terpos and coworkers (2) showed increased Sema4D in peripheral blood and bone marrow plasma from myeloma patients compared to controls. We have shown that myeloma cell lines and primary myeloma cells express Sema4D at higher levels than MDA-MB-131 cells. In addition, we found that coculture of myeloma cells with bone increased expression of Sema4D by both tumor cells and bone (84). Myeloma cells, as well as breast cancers, express MMP14 (MT1-MMP), which releases membrane-bound Sema4D by proteolytic cleavage (85). Sema4D can promote angiogenesis, which is required for myeloma progression. Sema4D is, therefore, a potential target in myeloma, as well as in cancers that metastasize to the skeleton.

## Strategy for SEMA4D/Plexin-B1 Targeting

VX15/2503 is the first and only currently available humanized Sema4D blocking antibody in clinical testing. The antibody was generated in *Sema4D*^−/−^ mice by using a panel of SEMA4D-specific hybridomas that react with murine, primate, and human SEMA4D (86). VX15/2503 bound with high affinity (1–5 nmol/L) to Sema4D and achieved complete Sema4D blockade in animal models, and its activity was subsequently confirmed in humans. No dose-limiting toxicity or maximal tolerated dose was observed at the dose required for complete Sema4D blockade in phase I clinical trials of multiple sclerosis and refractory solid malignancies (87, 88).

In the phase I study of 42 patients with refractory cancers receiving dose escalation of VX15/2503, one patient (2.4%) achieved partial response and 27 patients (64.3%) achieved long duration stable disease. Subjects with elevated baseline B and T lymphocytes exhibited longer progression-free survival, suggesting involvement of immune-mediated antitumor activity. Bone parameters and development of metastases were not endpoints. Tumor response was based on reduction in tumor size; therefore, patients with isolated bone metastases without other measurable tumor masses were not included.

The large, planar binding interface of the Sema4D/Plexin-B1 interaction makes it challenging to target with small molecules. Matsunaga and coworkers discovered a macrocyclic peptide, PB1m6, which binds Plexin-B1 with high affinity and specifically inhibits binding of the physiological ligand Sema4D and completely suppressed Sema4D-induced cell collapse *in vitro* (89). No data for this peptide *in vivo* or in cancer models are currently available.

Because of the central role of Sema4D in osteoblast inhibition, Zhang and group developed bone-specific drug delivery of *Sema4D siRNA* using *N*-(2-hydroxypropyl) methacrylamide copolymers with d-aspartic acid octapeptide (90). They showed that in an osteoporotic animal model induced by ovariectomy, weekly intravenous injections of this compound decreased osteoclast Sema4D expression and increased osteoblast differentiation. Treated animals showed higher femoral bone volume both in prevention and treatment models. Similar reduction of bone loss was seen in alveolar bone of the mandibles (91). Clinical testing of this molecule in patients has not been reported, but the study provides a proof of benefit for Sema4D targeting in bone.

While blocking the Sema4D/Plexin-B1 complex is of potential benefit in cancers and neuroinflammatory diseases, some cancers are growth inhibited by Sema4D signaling. In an acute myeloid leukemia cell line Kasumi-1, Sema4D binding of CD72, its preferred receptor in immune cells, leads to inhibition of growth and cell death, as a result of phosphorylation of CD72, the formation of the CD72–SHP-1 complex and dephosphorylation of src family kinases and JNK (15). Future development of Sema4D targeting should take into account the cellular context where Sema4D exerts its function.

## Discussion

We highlight Sema4D as a novel regulator of bone homeostasis. Sema4D also supports crucial steps in tumor progression, ranging from invasion, migration, angiogenesis, and immune suppression, to the pathological alteration of the tumor niche to support metastasis. Targeting Sema4D in the clinic has become practical with the development of a specific neutralizing antibody, which has low toxicity and an impressive response rate in early clinical trials of patients with refractory cancers. Because of the potential role of Sema4D in bone metastasis, this molecule should be further explored in these specific disease groups, and bone metastases should be included in clinical endpoints. Our preliminary data also support the potential of Sema4D blockade in multiple myeloma. Baseline B and T cell profiles have been correlated to response in a preliminary cancer clinical trial and should be further developed as a screening tool to identify patients likely to respond, or as a biomarker of response. In addition, Sema4D targeting may have synergistic antitumor effects when combined with immunomodulatory agents, warranting further study.

A major open question is whether Sema4D from non-osteoclast sources plays a significant role to inhibit osteoblast activity in cancer/bone diseases. Sema4D is expressed on tumor cells that colonize bone, while cells of the microenvironment other than osteoclasts may also express Sema4D. Although the extracellular domain can be proteolytically shed, it seems unlikely that local sSema4D concentrations are sufficient to activate osteoblast Plexin-B1. Both Negishi-Koga et al. (1) and Yang et al. (3) showed osteoblast suppression with micrograms per milliliter amounts of sSema4D:Fc fusions, orders of magnitude higher than those found in bone marrow plasma from multiple myeloma by Terpos et al. (2). Non-osteoblast and tumor sources of Sema4D could still inhibit osteoblast activity by direct cell:cell contact. Direct evidence for such actions is lacking; they may occur only in the context of co-receptors specific to the osteoclast. Patient data from clinical trials with Sema4D-neutralizing antibody could provide answers to these questions. Patients receiving antibody treatment should have increased bone mineral density. If the osteoclast Sema4D axis is the only significant contributor to osteoblast suppression, then there may be little bone effect of treatment in patients also receiving anti-osteoclast drugs (bisphosphonates or denosumab), which are the standard of care for cancer bone diseases.

## Author Contributions

All authors contributed to the writing of the manuscript.

## Conflict of Interest Statement

The authors declare that the research was conducted in the absence of any commercial or financial relationships that could be construed as a potential conflict of interest.
